# Queer in Chem: Q&A with Professor Andrew Goodwin

**DOI:** 10.1038/s42004-023-00972-9

**Published:** 2023-09-30

## Abstract

Andrew Goodwin is Professor of Materials Chemistry and a Professorial Research Fellow at the University of Oxford. His research focuses on the dual aspects of flexibility and disorder in functional materials, and his group of about 10–15 researchers is based in Oxford’s Inorganic Chemistry Laboratory.

**Figure:**
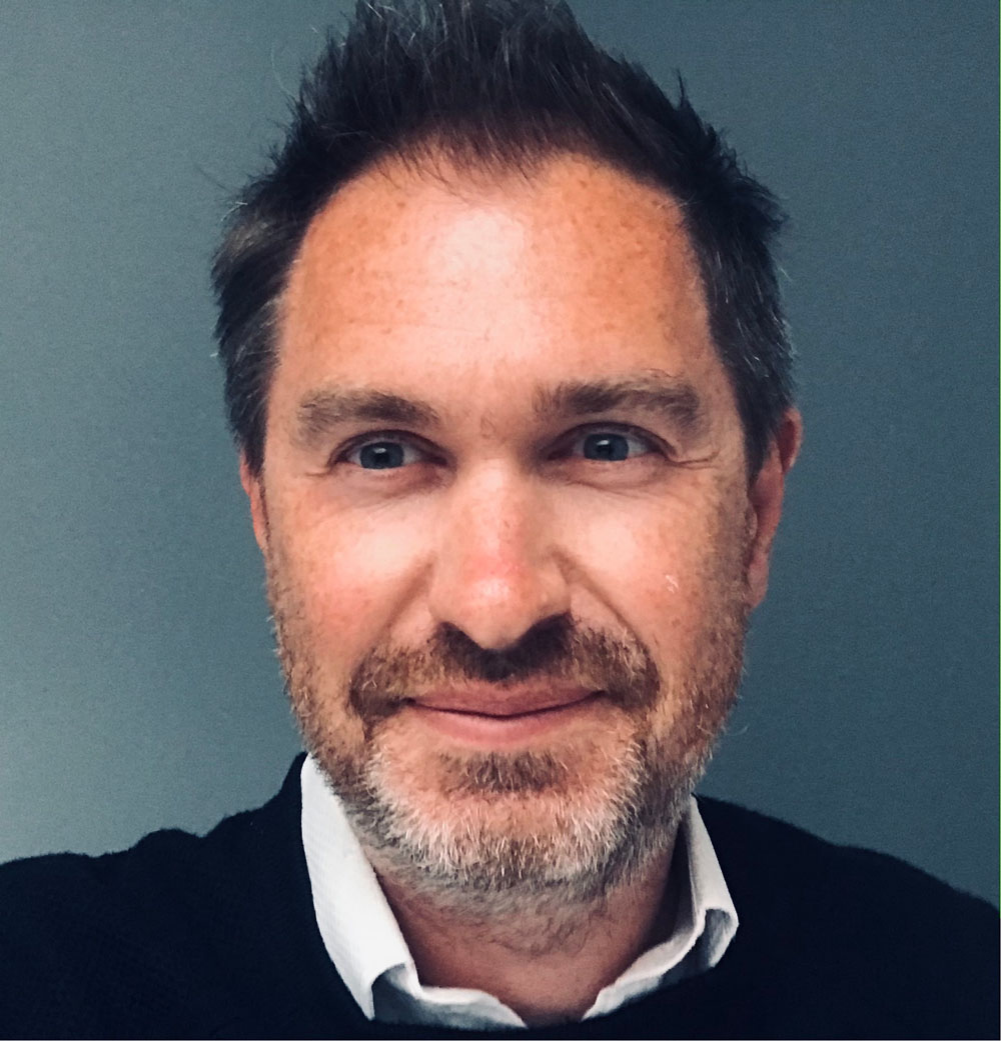
Andrew Goodwin

Andrew was born in Sydney, Australia, and studied at the Universities of Sydney and Cambridge. Following a Junior Research Fellowship in Materials Science at Trinity College, Cambridge, he joined the Chemistry Faculty at Oxford in 2009, first as a Lecturer, then Professor, and subsequently Research Professor. Andrew was the inaugural Chemistry Laureate of the Blavatnik Awards in the UK and was recently elected to a Fellowship of the Royal Society.

Why did you choose to be a scientist?

The easy answer is that I’m not much good at anything else! As a kid, I was always fascinated by how things worked, by geometry, and by numbers. No doubt, I was also influenced by the fact my father was a chemist—he even built me a rudimentary lab in the basement of our house. By the time I was doing my own research as a graduate student, I was utterly hooked. It’s an incredibly rewarding and varied job that I’m very lucky to have. I particularly value the mix of discovery, intellectual challenge, teaching, learning, and interaction with talented and creative people from many different walks of life.

What scientific development are you currently most excited about?

There are many, but let me use as an example the recent discovery of an ‘Einstein tile’. Our own research is focused on the phenomenology of disorder and flexibility in functional materials, and one of the key challenges in this field is developing an understanding of the kinds of complexity that can be present in materials on the atomic scale. The discovery of an Einstein tile—a relatively simple shape, copies of which can cover two-dimensional space completely, but only in ways that never repeat—is a reminder that Nature has many surprises up her sleeve. How exciting that there are fundamental aspects of structure that remain to be discovered, and exciting too to think of how these previously-unknown arrangements might translate into new and unusual materials properties.

What direction do you think your research field should go in?

I don’t presume to know in what scientific direction our field should develop: I think it’s important in materials chemistry, as in all domains, for individuals to follow their own sense of what constitutes an interesting direction to pursue. What I would say is that ours is a field so often focused on metrics—whether it’s the charge-storage capacity of battery materials, the conversion efficiency of solar cells, the figure of merit of thermoelectrics, or the strength of magnetoelectric coupling in multiferroics. Personally, I’m encouraged by what I perceive to be an increasing appreciation that the most profound advances can be conceptual in nature and are often rooted in quite fundamental principles of materials chemistry. We’re emerging from a phase of relatively heavy targeted investment into specific materials classes and materials applications, and I can see a greater role for unashamedly fundamental science alongside materials development as the field continues to mature.

How does your queer identity intersect with your identity as a scientist?

Obviously, a core aspect of growing up gay is a sense of feeling different to those around you. Does that sense of difference translate to thinking differently in a scientific context? That’s difficult to tell, but I do think—at least in my case—that the process of having dealt with being gay and coming out to friends and family can make one less afraid to challenge consensus. I’ve long considered myself to have a healthy disrespect for traditional subject boundaries, for example, and perhaps some of that unorthodoxy has its roots in an intersection between queer and scientific identities. A more straightforward way in which the two aspects meet is that I have been lucky to supervise a number of LGBTQ+ students, and I know that the diversity they help bring to the group is absolutely crucial in supporting a creative and inclusive environment in which to do excellent science.

Why do you think it is important to feel comfortable enough to bring your whole self to work?

Quite simply: people do their best work when they’re happy and accepted for who they are. Concealing a significant part of one’s own identity is an enormously difficult and emotionally draining process, and one that is entirely counterproductive. I absolutely do not buy the argument that one’s personal life should be left at home. Academic life involves forging relationships and collaborations that develop over decades, building a sense of trust and often quite genuine friendship. It would be entirely bizarre to avoid discussing one’s identity or personal relationships or family—in fact, doing so simply creates a barrier to interaction that inhibits collaboration. Having grown up in less accepting times than we enjoy now, I feel genuinely lucky that at this point in my career, here in the UK, I can be open about my sexuality. I can take my husband to professional events without fear of recrimination; I do not have to lie. And I’m sure that my science is better as a result.

How do you lift yourself and potentially the LGBTQ+ community up to thrive in chemical research?

As the question presumes, it probably will fall to us LGBTQ+ scientists both individually and collectively to do the lifting here. Personally, I think this comes down to three things. First is visibility. I do think it’s important that those amongst our community who feel comfortable doing so (not all will!) are visible as such in their scientific roles. For my own part, that might mean being open with my colleagues, students, and collaborators, or contributing an interview such as this, or writing openly about my experiences as a gay man in academia. Second is support. We need to help each other navigate academia, whether that’s through mentorship or calling out discriminatory behaviour or doing whatever we can to make our own environments as welcoming and supportive as possible. And third is quality. At the end of the day, no-one can argue with good science. If the professional advantage of feeling free to be open about one’s self is that we can be better scientists as a result, then we need to seize that opportunity with both hands.

*This interview was conducted by the editors of Communications Chemistry*.

